# Influence of different surgical techniques on primary implant stability in the posterior maxilla: a randomized controlled clinical trial

**DOI:** 10.1007/s00784-023-04962-y

**Published:** 2023-03-28

**Authors:** Maria-Victoria Olmedo-Gaya, María-Nuria Romero-Olid, Francisco M. Ocaña-Peinado, Marta Vallecillo-Rivas, Cristina Vallecillo, Candela Reyes-Botella

**Affiliations:** 1 Department of Stomatology, School of Dentistry, University of Granada, Granada, Spain; 2 Department of Statistics and Operations Research, University of Granada, Granada, Spain; 3 Faculty of Dentistry, University of Granada, Colegio Máximo de Cartuja s/n, 18071 Granada, Spain

**Keywords:** Dental implants, Implant stability, Bone quality, Conventional drilling, Underpreparation, Expanders

## Abstract

**Background and objective:**

Primary stability (PS) is remarkable for secondary stability and implant success. Surgical technique modifications seem to improve primary stability, especially in poor quality bone. The aim of this study was to compare the insertion torque (IT) and implant stability quotients (ISQ) of implants placed with underpreparation, expanders, and standard surgical instrumentation in different bone types.

**Material and methods:**

This randomized controlled clinical trial enrolled 108 patients (*n*=108 implants) distributed in three study groups: group 1 (*n*=36) underpreparation technique, group 2 (*n*=36) expander technique, and group 3 (*n*=36) conventional drilling. IT was recorded with a torque indicator. ISQ was recorded with resonance frequency analysis immediately after surgery.

**Results:**

ISQ values were associated with the patient’s bone quality and were higher in bone quality type II (76.65) and type III (73.60) and lower in bone quality type IV (67.34), with statistically significant differences (*p*<0.0001). Lower stability results were obtained when conventional drilling (69.31) was used compared to the use of underpreparation (74.29) or expanders (73.99) with a level of significance of *p*=0.008 and *p*=0.005, respectively.

**Conclusions:**

The surgical technique influences the PS when there is low-quality bone. In low-quality bones, conventional drilling obtains lower ISQ values.

**Clinical relevance:**

Replace the conventional drilling technique for an alternative, underpreparation or expanders, in low-quality bone in order to achieve greater primary stability.

## Introduction

Scientific evidence supports that dental implants achieve high survival rates and success, understood by the presence and maintenance of osseointegration and function (ability to chew) after being subjected to load [1]. Long-term prospective reports, with follow-up greater than 10 years, have shown implant survival rates of approximately 95% [2].

Ossteointegration was defined as a direct, structural, and functional connection between the bone and the surface of an implant subjected to functional loading [3]. This metabolic process is considered as a foreign body reaction with the formation of a direct interface between an implant and bone, without intervening soft tissue [4]. Studies have demonstrated that the absence of micromovements must occur during the process of biological union between both structures. According to Frost’s mechanostat [5], this control is necessary to avoid an increase in peri-implant bone microstress that can lead to bone loss, osseointegration failure, and implant loss [6]. It has been shown that movements greater than 150 millimicrons cause an undesirable interface at the bone-implant contact, which is not capable of supporting the functional load and eventually leads to implant failure [7, 8]. This concept of immobility at insertion or primary stability (PS) is therefore associated with the success of the bone healing or osseointegration process [9]. Moreover, the PS represents a necessary condition for secondary stability and for implant success [10].

Primary stability results from the mechanical interaction between the implant and the bony walls during the insertion [10, 11]. It has been shown that PS depends on several factors, including bone quality or density, the morphological characteristics of the implant, and the surgical technique used for insertion [6, 10]. The degree of implant stability can be subjectively measured by insertion torque (IT) values using surgical handpieces or objectively obtained implant stability quotients (ISQ) using resonance frequency analysis (RFA) [12]. Accepting the relationship between the interfacial stress distribution during implant installation and the respective strain of the peri-implant tissue due to frictional forces, these factors are considered as key morphometric predictors of primary stability [12]. Therefore, by modifying these factors, it will be possible to modify the PS.

In recent years, implantology has focused on designs that increase implant-to-bone contact introducing modifications in the macrostructure of the implants as well as modifying the surgical technique; however, some bone characteristics cannot be modify. According to Misch in 1989, bone quality can be classified into four types (D1–D4) based on macroscopic and trabecular characteristics [13]. D4 bone is highly trabecular and often lacks cortical bone. It is commonly found in the posterior maxilla, shows a Hounsfield Unit (HU) reading between 150 and 350 units, has the lowest implant-to-bone values, and has the highest failure rate of the four bone types [6]. Another used classification is the one proposed by Lekholm and Zarb [14]. In this classification, the volume and structural characteristics of the bone tissue are evaluated based on radiographs and the surgeon’s tactile perception regarding the hardness of the bone tissue [15]. Bone is classified based on a scale ranging from I to IV according to the amount of trabecular and cortical bone. In type 1, the bone tissue is composed almost entirely of cortical bone, while type 4 has areas of thin cortical bone around a main layer of trabecular bone [15]. It has been observed that PS is lower in type IV bone and implants inserted in type III–IV bones suffer higher failure rates (4.27–8.06%) than those placed in type I–II bones [16]. Various techniques have been used to improve the PS in type 4 bone. Site preparation for dental implant placement is a key step of all implant surgical procedures and implant survival [17, 18]. It has been shown that osteotomy drilling technique is a sensitive process with many fundamental associated factors for crestal bone stability and osseointegration of dental implants [17]. Friberg et al. [19] recommended the use of undersized drilling. Use of a final drill with a smaller diameter than that recommended by the manufacturer, leading to a smaller osteotomy, or underpreparation [19]. Summers proposed the use of a bone condensers or osteotomes after a pilot drill to displace the bone at the periphery of the cavity [20]. But not only the surgical technique can influence PS, but it also influences other clinical parameters that take place once the implant is placed and when its healing process begins, such as marginal bone loss (MBL). Antonacci et al. [21] performed a meta-regression based on the study of early MBL, which is the one that occurs during the first 6 months and is almost exclusively due to the surgical technique. In this way, these researchers demonstrate a significant trend towards a lower MBL when conventional drilling is performed compared to the underpreparation technique [21]. Underpreparation techniques could cause overheating or excessive compression of the cortical bone with the formation of microfractures [22]. The aforementioned authors recommend carefully selecting the drilling sequence based on bone density to achieve optimal primary stability and preserve crestal bone morphology [21]. In this way, it is important that the clinician knows how the surgical technique influences not only the PS but also all those factors that intervene in the success of implant therapy [17, 18, 23]. It has been verified that a wrong choice of surgical technique can lead to what Zucchelli et al. [23] classified as technical related-risk factors and operator-related complications and even failure (early or late) of implants [18]. Therefore, taking into account that success of implants is based primarily on the preservation of bone support and its stability [24], depending on the type of bone density, the surgeon is able to decide whether to modify conventional drilling for another technique.

This randomized clinical trial (RCT) aimed to compare the PS (immediate after implant placement) of sites prepared with underpreparation, expanders, and standard surgical instrumentation at different posterior maxillary bone types. The postulated null hypothesis was that surgical technique would not influence on implant primary stability measured as IT values and ISQ values after implant placement especially in bone type IV.

## Material and methods

### Study design and patient selection

This study was design as single-center, double-blind RCT to study the influence of surgical technique on PS in different bone types. This trial was conducted in patients undergoing the placement of dental implants in maxillary second premolars and molars sites at the Clinic of the School of Dentistry of the University of Granada (Spain) between January 2021 and March 2022.

All participants received a detailed description of the study protocol and signed informed consent to participate in the study, which followed the guidelines of the Helsinki Declaration and was approved by the ethics committee (n° 2324/CEIH/2021) of the University of Granada. This clinical trial was registered in the Australian New Zealand Clinical Trials Registry (ACTRN12622001311741) and follows the recommendations of the CONSORT 2010 statement for reporting randomized trials [25, 26].

Study inclusion criteria were as follows: age ≥18 years, ASA I–II status according to the American Society of Anesthesiologists, edentulism in the maxillary second premolar or molar region, smokers of less than 10 cigarettes/day, periodontally healthy or controlled periodontal disease, and patients who sign the consent to participate in the study. The exclusion criteria were as follows: age under 18 years, pregnancy or breast-feeding, ASA III, IV, or V status (diabetes uncontrolled, heart diseases, bleeding disorders, compromised immune system), previous or active treatment with antiresorptive drugs (denosumab/bisphosphonates), use of illicit drugs, irradiated patients, severe psychiatric illness, and poor oral hygiene or untreated periodontal pathology.

### Sample size

Using ISQ values as the objective variable of the study and assuming an overall standard deviation of all implants less than 7.5 units based on previous studies [27], the sample size was calculated for 33 subjects per treatment. This provides 90% power to detect a mean difference between treatments of at least 3.5 units. Considering the possibility of losing 15%, a final theoretical sample of 36 individuals was calculated for each treatment.

A total of 108 were consecutively admitted in the study following a scheme of balanced randomization using a computer-generated randomization sequence. Each participant was randomized assigned (1:1 ratio) to one of the 3 treatment groups, so 36 implants were placed with the underpreparation technique (group 1), 36 implants with the expander technique (group 2), and 36 implants using conventional drilling (group 3).

### Material

The implants used for this RCT were BTI® implants (Biotechnology Institute, Vitoria, Spain). BTI® internal implants are implants with parallel walls and conventional conical apex with internal connection of tetralobe design. They present a UnicCa® surface, which is a chemical modification with calcium ions on the triple nanoroughness. The implants presented different diameters and lengths. For the preparation of the bone bed with the expander technique, motorized expanders of the BTI® brand (Biotechnology Institute, Vitoria, Spain) were used. Primary stability was measured by RFA, using the Osstell® IDx device (Osstell®, Gothenburg, Sweden) to measure ISQ values.

### Surgical protocol

All patients eligible to participate in the study underwent a detailed clinical examination and exhaustive radiological study consisting of a CBCT in order to three-dimensionally assess the relationship with important anatomical structures and bone availability and quality. For implant placement, the socket should be completely healed, being a type IV socket according to the classification of Tonetti et al. 2019 [28]. Once the indication for placement of an implant in the molar and second premolar region was determined, the patient was randomly assigned to one of the three groups and given an appointment in the operating room of the Faculty of Dentistry of the University of Granada. All interventions were performed by the same operator (MVOG). Immediately before the intervention, patients rinsed their mouths for 2 min using 10 mL of 0.12% chlorhexidine mouthwash (Perio-Aid®; Dentaid®, Barcelona, Spain), and received local anesthesia using 4% articaine with 1:100,000 epinephrine (Ultracain®; Normon®, Madrid, Spain). A full-thickness ridge incision was made to prepare the bone bed. At this time, the sealed envelope was opened with the assignment of the patient to perform one of the three study interventions. Patients in group 1 underwent an underpreparation technique using a final drill with a smaller diameter than the recommended by the manufacturer according to bone quality (Table 1). The implant bed preparation in group 2 was prepared with motorized bone expanders. When the motorized bone expansion technique was used, the procedure for the insertion of the implants began with a lanceolate drill followed by the introduction of the expanders and ending with the expander indicated by the manufacturer according to the final diameter of the implant to be placed, as indicated in Table 1 and at 30 rpm and with no irrigation. The selection of the expander was made according to the expander recommended by the manufacturer for each of the different diameters. In group 3, a conventional drilling technique was performed following the manufacturer’s instructions according to bone quality (Table 1). The implants were placed without bone reduction, remaining inserted at the crestal level. The IT was determined at the time of implant placement. Thanks to the motor torque control, it was possible to measure the torque at which the implant was placed, also known as compression torque [21]. Once the implant was placed, a smartpeg number 27 was used and a RFA was performed using Osstell® IDx to measure PS of the implant. All the implants were placed by the same surgeon (MVOG) and the PS of the implants was measured by someone other than the surgeon, always the same (CRB), who was unaware of the surgical technique used for their insertion. The incision was sutured to close the wounds with 3.0 silk suture (Normon®).

**Table 1:** Surgical drilling protocols for implant placement in underpreparation, conventional drilling, and expanders technique

Bone quality	Implant diameter	Final drill		
		Underpreparation	Conventional	Final expander
II	3.3 mm	1.8/2.5 mm	2.8 mm	II (2.6 mm)
	3.75/4.0 mm	3.25 mm	3.5 mm	III (3.1 mm)
	4.5 mm	3.75 mm	4.0 mm	IV (3.8 mm)
III	3.3 mm	1.8 mm	1.8/2.5 mm	II (2.6 mm)
	3.75/4.0 mm	3.0 mm	3.25 mm	III (3.1 mm)
	4.5 mm	3.5 mm	3.75 mm	IV (3.8 mm)
IV	3.3 mm	Initial	1.8 mm	II (2.6 mm)
	3.75/4.0 mm	2.8 mm	3.0 mm	III (3.1 mm)
	4.5 mm	3.25 mm	3.5 mm	IV (3.8 mm)

Patients were prescribed with a 0.12% chlorhexidine mouthwash (Perio-Aid®; Dentaid®) to use after tooth-brushing for 1 week. All patients were instructed to follow a soft and tepid diet in the first 3 days after surgery, along with instructions for oral hygiene. They received a prescription for Amoxicillin 750 mg (or 300 mg clindamycin for penicillin-allergic patients), one tablet every 8 h for 5 days. Additional prescriptions included anti-inflammatory and analgesic drugs for 3 days.

### Study variables

Study variables were classified into patient, implant, and surgical technique variables.

Patient’s variables were as follows: age, sex, implant position (second premolar/first molar/second molar), and bone quality (I/II/III/IV) according to the Lekholm and Zarb bone density criteria determined by resistance to drilling during socket preparation and considering the CBCT [14].

Implant’s variables collected were length (≤8.5mm/>8.5mm) and diameter (narrow=3.3mm/standard=3.75–4mm/wide=4.5mm).

The intervention variables were surgical technique used (underpreparation/expanders/conventional drilling) and insertion torque (<40 Nw/≥40 Nw) considered as secondary outcome variable. Finally, the initial ISQ was collected as primary outcome variable.

### Statistical analysis

Statistical analysis was carried out using SPSS v.26 software. Quantitative variables were summarized by mean and standard deviation (SD), and for qualitative variables, percentages were used. The normality of the variables was studied using the Shapiro-Wilk test. The hypothesis of equality of variances was checked using the Lévene test.

Student’s *t* test or the Mann-Whitney test was applied, as appropriate for bivariate tests. The association between qualitative variables was studied using the chi-square test (applying Fisher’s correction in the case of 2×2 tables, together with measures of risk, OR, and relative risk). In the multivariate analysis, an analysis of variance of two factors was applied considering the interaction between both. For multiple comparisons, the Bonferroni adjustment was applied. The level of significance, *α*, was set at 0.05 in all cases.

## Results

### Descriptive analysis

One hundred and eight patients were included in the study to be placed 108 implants and randomly assigned to one of three study groups. Group 1 (*n*=36) underpreparation technique, group 2 (*n*=36) expander technique, and group 3 (*n*=36) conventional drilling. Two patients were lost to the follow-up, one of them belonged to group 1 and the reason for the loss was the patient’s refusal to continue participating in the study. The second patient who was lost belonged to group 3 and was excluded because the implant did not achieve sufficient stability to be able to measure it with the Osstell®. The final study sample therefore comprised 106 implants placed in 47 men (44.3%) and 59 women (55.7%). The mean age of the patients was 55.35±11.80. Most of the implants were placed in the first molar position (49.06%) and in bone quality type III (47.20%) and IV (37.7%). Regarding the variables of the implants, the most frequent length was >8.5 mm (73%) and the most used diameter was 3.75/4 mm (76%). The average primary ISQ value was 72.44 ± 6.31. Table 2 summarizes results obtained for the study variables.

**Table 2:** Summary of patient, implant, and bone data

		Implants	
		*(n)*	(%)
Sex	Male	47	44.3
	Female	59	55.7
Implant length	≤ 8.5 mm	33	31.1
	> 8.5 mm	73	68.9
Implant diameter	3.3 mm	8	7.55
	3.75/4.0 mm	76	71.70
	4.5 mm	22	20.75
Implant location	Second premolar	49	45,28
	First molar	51	49.06
	Second molar	6	5.66
Insertion torque	< 40 N	40	37.7
	≥ 40 N	66	62.3
Bone quality^*^	II	16	15.1
	III	50	47.2
	IV	40	37.7

*Lekholm and Zarb classification

### Clinical parameters

Potentially influential variables in the ISQ were studied. The diameter (*p*=0.392) and the length (*p*=0.237) of the implants were not associated with the initial ISQ.

ISQ values were associated with the patient’s bone quality and were higher in bone quality type II (76.65) and type III (73.60) and lower in bone quality type IV (67.34), with statistically significant differences (*p*<0.0001). Although higher values were obtained in type II bone than in type III, no statistically significant differences were reached. The mean ISQ values associated with bone quality are shown in Table 3.

**Table 3:** Postoperative ISQ values related to bone quality

*a. Estimations*				
Bone quality	*n* (mean)	SE	95% CI	
II	16 (76.65)	1.42	(73.84, 79.47)	
III	50 (73.60)	0.78	(72.06, 75.14)	
IV	40 (67.34)	0.95	(65.46, 69.22)	
*b. Pairwise comparisons*				
Bone quality	Difference of means	SE of difference	95% CI	*p*-value
II–III	3.05	1.62	(−0.89, 6.99)	0.186
II–IV	9.31	1.70	(5.16, 13.47)	0.000
III–IV	6.26	1.22	(3.28, 9.24)	0.000

*CI* confidence interval, *SE* standard error

The relationship of ISQ values with the surgical technique used was also studied. Lower stability results were obtained when conventional drilling (69.31) was used compared to the use of underpreparation (74.29) or expanders (73.99) with a level of significance of *p*=0.008 and *p*=0.005, respectively. The mean ISQ values associated with the surgical technique are shown in Table 4.

**Table 4:** Postoperative ISQ values related to the surgical technique

*a. Estimations*				
Surgical technique	*n* (mean)	SE	95% CI	
Underpreparation	35 (74.29)	1.20	(71.90, 76.68)	
Expanders	36 (73.99)	0.94	(72.13, 75.85)	
Conventional	35 (69.31)	1.09	(67.16, 71.47)	
*b. Pairwise comparisons*				
Surgical technique	Difference of means	SE of difference	95% CI	*p*-value
UND-EXP	0.30	1.53	(−3.42, 4.02)	1.000
UND-CONV	4.98	1.62	(1.03, 8.93)	0.008
EXP-CONV	4.68	1.44	(1.18, 8.17)	0.005

*CI* confidence interval, *CONV* conventional, *EXP* expanders, *SE* standard error, *UND* underpreparation

**Fig. 1:**
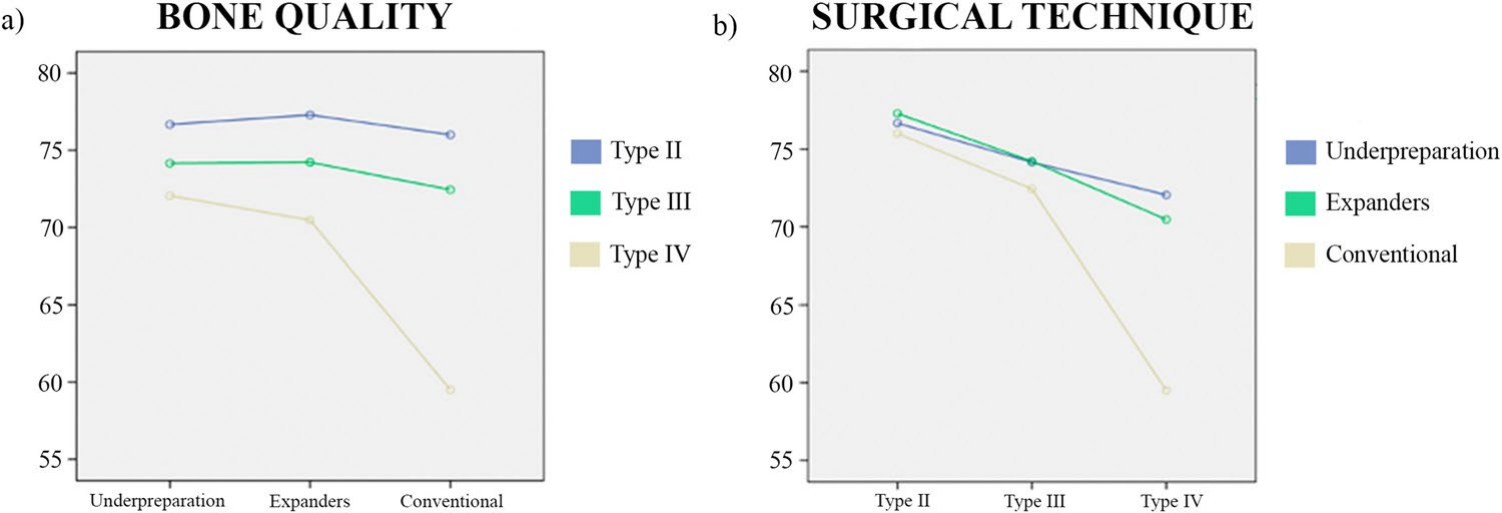
ISQ values in relation to **a** surgical technique for the different qualities of bone and **b** bone quality for the different surgical techniques used

Although no significant differences were obtained between the underpreparation technique and osteotomes in terms of the initial ISQ values, when the implants were placed in low-quality bone, the best results were obtained with the underpreparation technique (Table 5) (Fig. 1).

**Table 5:** Postoperative ISQ values as a function of bone quality and the surgical technique

*a. Estimations*					
Bone quality	Surgical technique	*n* (mean)	SE	95% CI	
II	Underpreparation	3 (76.67)	3.06	(70.59, 82.75)	
	Expanders	7 (77.29)	2.01	(73.31, 81.27)	
	Conventional	6 (76.00)	2.17	(71.70, 80.30)	
III	Underpreparation	13 (74.15)	1.47	(71.23, 77.08)	
	Expanders	14 (74.21)	1.42	(71.40, 77.03)	
	Conventional	23 (72.44)	1.11	(70.24, 74.63)	
IV	Underpreparation	19 (72.05)	1.22	(69.64, 74.47)	
	Expanders	15 (70.47)	1.37	(67.75, 73.19)	
	Conventional	6 (59.50)	2.17	(55.20, 63.80)	
*b. Pairwise comparisons*					
Bone quality	Surgical technique	Difference of means	SE of difference	95% CI	*p*-value
II	UND-EXP	−0.62	3.66	(−9.54, 8.30)	1.000
	UND-CONV	0.67	3.75	(−8.48, 9.81)	1.000
	EXP-CONV	1.29	2.95	(−5.91, 8.48)	1.000
III	UND-EXP	−0.06	2.04	(−5.04, 4.42)	1.000
	UND-CONV	1.72	1.84	(−2.77, 6.21)	1.000
	EXP-CONV	1.78	1.80	(−2.60, 6.16)	0.975
IV	UND-EXP	1.59	1.83	(−2.88, 6.05)	1.000
	UND-CONV	12.55	2.49	(6.50, 18.61)	0.000
	EXP-CONV	10.97	2.56	(4.72, 17.21)	0.000

*CI* confidence interval, *CONV* conventional, *EXP* expanders, *SE* standard error, *UND* underpreparation

The underpreparation technique showed good ISQ values regardless of bone quality. The expander technique obtained lower ISQ values in low-quality or type IV bone compared to type II bone. The conventional drilling technique recorded lower stability values in type IV bone, followed by type III and better stability values in type II bone.

The association of IT with other qualitative variables found a significant association of this variable with the type of bone (*p*<0.0001). No significance were found for the surgical technique used (*p*=0.268), the diameter (*p*=0.716), and length (*p*=0.832).

Data on insertion torque and bone quality are shown in Table 6. There was only 1 implant placed in type II bone with a torque <40 Nw, which indicated that higher insertion torque values were found in type II bone. For this reason, only type III and type IV bone have been considered in the association study.

**Table 6:** Insertion torque and bone quality of the implants

Bone quality	Insertion torque	Implants (*n*)	%
III	< 40 N	12	13.3
	≥ 40 N	38	42.2
		50	55.6
IV	< 40 N	27	30.0
	≥ 40 N	13	14.4
		40	44.4
Total	< 40 N	39	43.3
	≥ 40 N	51	56.7
		90	100

In relation to bone type, type III was associated with the highest insertion torque and type IV with the lowest insertion torque, OR = 0.152, 95% C.I. OR = (0.060, 0.384). Within the group of implants with torque lower than 40Nw, we have RR = 0.356, 95% C.I. RR = (0.208, 0.609). Therefore, it is less likely to have torque lower than 40Nw in type III bones than in type IV bones.

## Discussion

The present study has been carried out to analyze the influence of the surgical technique on the primary implant stability. The results found support that dental implants placed after conventional drilling obtained lower ISQ values than those placed with underpreparation techniques or the use of expanders in poorer quality bones.

In the literature, it has been shown that torque, ISQ, and therefore PS depend on the quality of implant-bone contact in the cortical part and this is influenced by the surgical technique used, the macroscopic design of the implant, and bone quality [6, 10, 29]. There are many studies that continue examining the clinical parameters that most influence the PS of implants. However, many of the published studies are experimental, ex vivo, in vitro, and only a few have a clinical trial design. Our RCT was performed according to CONSORT guidelines, restricting variables such as implant location and implant type. Even so, the sample size is large (108 patients) and sufficient to discriminate between the categories.

Regarding the relationship between bone quality and primary stability, our results show that higher ISQ values were associated with type II and III bone quality and lower ISQ values with type IV bone quality, according to several authors. Several works have shown that bones with more cortical density and less medullary component have higher ISQ values [30, 31]. In accordance with Anil et al. [32], when compared the PS of implants in different bone qualities, statistically higher ISQ values were observed for implants inserted in type II bone with 1-mm cortical bone respect to type IV bone quality. Nonetheless, in our study, statistical differences between type II and III bones were unable to detect. In accordance with Anitua et al. [33], type II bone is described with a density of 850–1000HU, composed of 3–4-mm-thick cortical bone surrounding a dense cancellous bone and corresponds to Lekohlm and Zarb type II bone. Type III bone presents a density of 550 to <850HU, composed of 2-mm-thick cortical bone surrounding a dense cancellous bone and corresponds to Lekohlm and Zarb type III bone [33]. Therefore, the absence of significant differences between ISQ values in type II and III bone could be due to the limitation of the present study, in which bone density was determined by CBCT and surgical tactile sensation. CBCT grayscale bone density values are not absolute, as in conventional CT. However, Arisan et al. [34] admitted that they can be predictive to measure bone quality subjectively. In the same way, a continuous variable such as bone density in an imaging test does not sometimes allow us to achieve significance, as it depends on a wide range of measurements. Currently, it has been shown that the most accurate way to establish bone density is by CT, since it is the only radiographic device that provides a numerical value that allows for objective measurement of bone density in HU units, while currently CBCTs no longer provide HU, and their use to provide information on bone density was questioned [35, 36]. As Anitua et al. [33], we used cone beam computed tomography to measure bone density. In addition, to complete this measurement we determined the bone density during surgical drilling.

In the present study, it has also been possible to verify the relationship between the ISQ values and the surgical technique used for the placement of the implants. Higher stability results have been obtained when underpreparation or expanders are used compared to conventional drilling, especially in cases of lower bone density. These results are consistent with two RCTs [37, 38] that concluded that PS in soft bone tissue was greater with the utilization of bone condensing osteotomes rather than conventional burs, regardless of the implant macrodesign [37]. Furthermore, we agree with the aforementioned authors that implant macrodesign does not strongly influence PS, since our results did not achieve significance in diameter or length variables of the implant either. This has been observed by other authors such as Akca et al. [39], who reported that bone quality had more influence than implant shape. This could be explained considering that PS depends on implant-bone contact in the cortical part, so length should not be a factor that influences ISQ [40, 41]. More authors have compared different techniques and have related them to the macrodesign of the implant. Specifically, Markovic et al. [37] found greater PS in self-tapping implants versus non-self-tapping tapered implants, but only when placed by conventional drilling, since this difference disappeared when the bone condensing technique was applied. This may suggest a relationship between design and surgical technique, which would modify the bone density and in turn influence the stability of the implant. On the contrary, Falisi et al. [42] observed no significant differences when comparing 5 techniques, including underpreparation, the use of motorized expanders, and conventional reaming. This can be explained because these authors previously used acid-treated pork ribs to achieve type IV density and were not performed on patients. On the other hand, and like us, Tabassum et al. [43] support that lateral and axial compression improved the primary-implant-stability and therefore this new surgical-technique should be considered as an alternative approach especially for placing implants in low-density bone. This finding were tested in earlier studies, performed on synthetic-bone-blocks [44, 45] and animal studies [46, 47]. Furthermore, Lemos et al. [48] also achieve higher PS results when they use underpreparation, although their study is performed only on fresh density III bone of bovine origin. In a similar study, Herrero-Climent et al. [49] also coincide with our study regarding an increase in TI and ISQ, although it is again a study carried out on fresh type III bovine bone. Our study offers the advantage of being carried out in human patients and in different types of bone, which, added to all the previous results, allows us to conclude that the surgical technique is one of the most influential factors in primary stability. In addition, the surgical technique makes it possible to compensate for the low bone density in the area that receives the implant. From the biomechanical standpoint, an undersized drilling protocol is effective in increasing insertion torque in low-density bone [22]. Nevertheless, it has been seen that these differences in PS between techniques disappear when bone quality is high. In dense cortical bones, compaction techniques do not offer improvements in PS measured by IT and ISQ. This could mean that the surgical technique influences when bone quality is compromised, there being a density “ceiling” above which alterations in the technique do not offer higher ISQ values. Therefore, it could be argued that underpreparation allows us to obtain greater stability results in low-quality bone, type IV, and even on certain occasions in type III. However, in those cases where the bone presents an acceptable quality, types I and II, this technique does not seem to provide any benefit, but it can also cause a compromise in the vascularization of the bone [21, 50]. In this sense, Antonacci et al. [21] describe in a systematic review and a meta-regression study a limit on the IT that should not be exceeded (50 to 70 Ncm) due to the occurrence of microfractures, microcirculation failure, and a tendency to necrosis. In our study, SSI improvements were produced only in low-density bones. The drilling sequence based on these results must be carefully chosen to preserve the integrity of the bony cortex while obtaining the best primary stability.

This study has corroborated by RCT that there is a relationship between bone quality and PS. Secondly, it has shown that stability in terms of ISQ improves in certain types of bone by modifying the surgical technique. The clinical application of these results could be that once the bone density of the receptor area has been radiographically determined, we can predict the torque that will reach our implant if we perform conventional drilling. Therefore, if we detect a low bone quality, we can modify our surgical procedure and opt for an alternative technique that provides us with a higher final ISQ. The alternative bed preparation techniques to conventional drilling proposed are the use of motorized expanders and underpreparation. These techniques, highlighting underpreparation, significantly increase the primary stability of an implant when placed in low-density bone.

## Conclusions

Bone quality has influenced both IT and ISQ values. PS have been modified by surgical technique in low-quality bone. In low-quality bones, conventional drilling obtained lower ISQ values.

## Data Availability

The data presented in this study are available on request from the corresponding author.
